# The utility of stereotactic biopsy of intracranial lesions in the diagnosis of leukemia complicated by central nervous system lesions

**DOI:** 10.1186/s41016-025-00392-9

**Published:** 2025-03-19

**Authors:** Xiaolong Wu, Yiqiang Zhou, Leiming Wang, Feng Yan, Huaqiang Zhang, Xiaotong Fan, Penghu Wei, Yongzhi Shan, Yaming Wang

**Affiliations:** 1 Department of Neurosurgery, Xuanwu Hospital Capital Medical University, Beijing, 100053 China; 2 International Neuroscience Institute (China-INI), Beijing, China; 3 Department of Pathology, Xuanwu Hospital Capital Medical University, Beijing, 100053 China

**Keywords:** Stereotactic Biopsy, Leukemia, CNS lesions, Pathology

## Abstract

**Background:**

Leukemia complicated by central nervous system (CNS) lesions (LCNSL) includes leukemia involving the CNS (CNSL) and CNS secondary lesions related to leukemia treatment (e.g., CNS infections, leukoencephalopathy, inflammatory demyelination, and vascular diseases). The clinical manifestations and imaging characteristics of different types of LCL are similar, increasing the possibility of misdiagnosis. This study aimed to enhance our understanding and management of LCL.

**Methods:**

We retrospectively collected clinical data from 22 patients with LCL and analyzed their magnetic resonance imaging and pathological characteristics. Pathological diagnoses were made using stereotactic intracranial puncture biopsy.

**Results:**

Between April 2003 and December 2023, 22 patients with LCL were admitted, including 18 males and 4 females aged 7–71 years. Bone marrow aspiration identified 14 cases of acute lymphoblastic leukemia (ALL), one of chronic lymphoblastic leukemia, six of acute myeloid leukemia (AML), and one of chronic myelomonocytic leukemia (CMML). Most patients presented with non-specific symptoms, including headache, nausea, vomiting, limb convulsions, and changes in mental status. A few patients had localized neurological deficits, such as limb weakness and blurred vision. Common systemic symptoms included fever, night sweats, and weight loss. The pathological diagnoses of the 22 patients were CNSL in 13 patients, CNS infections in five patients, and neurodegenerative diseases in four patients. Discrepancies were found between the clinical and pathological diagnoses in eight cases.

**Conclusions:**

Stereotactic intracranial lesion biopsy is minimally invasive, safe, convenient, and critical in the early and differential diagnosis of LCL. Early identification of the lesions’ nature and timely implementation of accurate and precise treatments can improve patient prognosis.

## Background

Leukemia is a malignant clonal disease of hematopoietic stem cells and a primary malignant tumor of the blood. In China, the incidence rate of leukemia is 8.34–10.87 per 100,000, ranking high among cancer mortalities [1–3]. Factors such as difficulty in treatment and high treatment costs severely affect patients with leukemia and their families [2]. The continuous optimization of treatment methods has increased the survival time of patients with leukemia. However, because chemotherapeutic drugs have difficulty crossing the blood–brain barrier (BBB), the CNS becomes a “sanctuary” for leukemia cells, increasing the incidence of CNSL [1–3]. Secondary CNS lesions related to leukemia treatment include CNS infections, leukoencephalopathy, inflammatory demyelination, vascular diseases, and secondary tumors following leukemia treatment [1, 3]. LCL includes CNSL and secondary CNS lesions related to leukemia treatment. The clinical manifestations and imaging characteristics of different types of LCL are similar, increasing the possibility of misdiagnosis [4–6]. Although CSF morphological examination has high specificity, its sensitivity is relatively low, leading to false-negative results. Flow cytometry improves diagnostic accuracy but is limited by the number of antibodies in CSF and has not yet been widely adopted [7–9]. Therefore, relying solely on conventional diagnostic methods is insufficient for accurately identifying CNSL, and a more precise pathological examination is necessary to improve diagnostic accuracy. Although clinical manifestations and imaging features share similarities, the treatment principles differ greatly; therefore, early and accurate diagnosis is important for efficacious treatment [10–12]. Stereotactic biopsy is the best method for obtaining pathological tissue from intracranial lesions, is minimally invasive, and enables precise localization [13–15]. Intracranial hemorrhage is the most common serious complication of stereotactic brain biopsy, with a reported incidence of approximately 0.5%–3% in previous studies. However, with advancements in imaging technology and surgical techniques, the safety of stereotactic brain biopsy has significantly improved [16, 17]. For lesions that remain unclear based on clinical manifestations and imaging features alone, stereotactic biopsy can facilitate a definitive pathological diagnosis, which is essential for formulating the subsequent treatment plan [13, 15].

This study analyzed the magnetic resonance imaging (MRI) and pathological features of 22 patients with LCL (all underwent stereotactic intracranial biopsy for a definitive pathological diagnosis). This study aimed to explore the use of stereotactic intracranial biopsy in diagnosing leukemia with CNS lesions to facilitate early and accurate diagnosis and timely and effective treatment.

## Methods

### Patient selection

We collected information on patients with LCL treated in our department between April 2003 and October 2022. The data included clinical manifestations, imaging features, and pathological diagnostic characteristics of CNS lesions. The inclusion/exclusion criteria were: (1) a confirmed diagnosis of leukemia, (2) the presence of a space-occupying lesion in the central nervous system, and (3) a definitive pathological diagnosis obtained via stereotactic biopsy.

## Preoperative imaging and intracranial stereotactic brain biopsy

Two neuroradiology experts independently evaluated the cranial imaging examination data including computed tomography (CT), MRI, and positron emission tomography (PET). The assessments focused on the lesions’ location, size, boundaries, and the extent of surrounding edema.

All 22 patients underwent stereotactic intracranial lesion biopsy to obtain pathological diagnostic results [14]. The framed stereotactic intracranial lesion biopsy procedure involved several steps, including attaching a head frame, imaging scans, target calculation, biopsy, pathological examination, and postoperative management [14]. The frameless stereotactic intracranial lesion biopsy procedure included marking with a surgery-specific robot marker, imaging scans, image fusion, delineating the lesion, determining the target point, designing the path, registering the markers, optimization assessment, biopsy, pathological examination, and postoperative management [6, 14, 18–20].

## Results

### Baseline and clinical data

Between April 2003 and December 2023, 22 patients with LCL were admitted, including 18 males and 4 females aged 7–71 years. Bone marrow aspiration identified 14 cases of acute lymphoblastic leukemia (ALL), one of chronic lymphoblastic leukemia, six of acute myeloid leukemia (AML), and one of chronic myelomonocytic leukemia (CMML). Most patients presented with non-specific symptoms, including headache, nausea, vomiting, limb convulsions, and changes in mental status. A few patients had localized neurological deficits, such as limb weakness and blurred vision. Common systemic symptoms included fever, night sweats, and weight loss. The details are presented in Table 1.

**Table 1:** Baseline and clinical features of patients with Leukemia complicated by central nervous system lesions

Case	Age	Gender	Leukemia	Clinical Manifestations	Imaging Features	Clinical Diagnosis	Pathological Diagnosis
1	15	M	AML(M5)	Seizures	Multiple cystic-solid lesions in the right frontal lobe and left occipital lobe, with enhanced cyst walls. Diffusion imaging shows high signal intensity within the lesions	Brain abscess	Brain abscess (Fungal)
2	20	M	ALL	Headache, Vomitig, Seizures	Multiple nodular lesions with long T1 and long T2 signals in the cortex and subcortex of both cerebral hemispheres, with unclear boundaries. There is evident perilesional edema. Enhancement scan reveals multiple nodular enhancements resembling "small cysts"	CSNL	Brain abscess (Fungal)
3	39	M	ALL	Headache, Vomiting, Lethargy	Abnormal mixed patchy signal adjacent to the right temporal horn of the lateral ventricle, with mixed T1 and long T2 signals, surrounding brain tissue edema, partial enhancement. Additionally, multiple small circular lesions adjacent to the left posterior horn of the lateral ventricle, exhibiting small volume, short T1 and long T2 signals, surrounded by brain tissue edema, and ring enhancement	Neurodegenerative disease	Brain abscess (Fungal)
4	26	F	AML (M4)	Fever, Headache, Slurred speech, Mouth drooping, Seizures, Loss of consciousness	Mixed signal intensity at the junction of the left frontal, temporal, and parietal lobes, measuring 4.3 × 4.5 cm, with prominent enhancement of the lesion and surrounding brain tissue edema	CSNL	Brain abscess (unknown pathogen)
5	43	F	ALL	Headache	Prominent enhancing mass lesion in the right frontal lobe, measuring 3.1 × 3.5 cm, accompanied by significant edema in the surrounding brain tissue	CSNL	Tuberculoma
6	49	M	ALL (B cell)	/	Enhanced lesions with surrounding edema in the left parietal and occipital lobes, and a softening lesion in the right parietal lobe	CSNL	Gliosis
7	71	M	AML	Sudden onset of right-sided limb movement impairment	The lesion in the right frontal lobe demonstrates long T1 and long T2 signals, with prominent ring enhancement on contrast-enhanced scans, but no internal enhancement	CSNL	Gliosis
8	60	M	CLL (B cell)	Limb weakness, unsteady gait	Patchy abnormal signals in the left pontine and cerebellar hemispheres, with scattered patchy abnormal signals around the bilateral lateral ventricles, demonstrating short T1 and long T2 signals without mass effect. Partial enhancement is observed in the lesions of the left pontine and cerebellar hemispheres	Neurodegenerative disease	Demyelinating lesion
9	23	F	ALL (B cell)	Headache	Lesions with long T1 and long T2 signals are observed in the right lateral ventricular frontal horn white matter, with unclear boundaries. There is no significant edema, and no enhancement is seen on contrast-enhanced scans	Neurodegenerative disease	Demyelinating lesion
10	22	M	ALL(B cell)	Dizziness with double vision, Vomiting, Unsteady gait	Abnormal patchy or nodular signals are observed in the left medulla oblongata, pons, midbrain, bilateral cerebellar hemispheres, bilateral thalami, splenium of the corpus callosum, periventricular regions, cortical and subcortical areas of the right frontal and parietal lobes, and left parietal and occipital lobes. These lesions show significant enhancement	Neurodegenerative disease	B-cell lymphoblastic leukemia involving CNS
11	7	M	ALL (B cell)	Headache, Dizziness	Cystic mass lesion in the right temporal-occipital region, with surrounding brain tissue edema	Brain abscess	B-cell lymphoblastic leukemia involving CNS
12	54	M	ALL (B cell)	Headache, Limb numbness, Vomiting, Seizures	Patchy enhancing lesions are visible in the posterior parts of both parietal lobes, and the meninges demonstrate "gyriform" enhancement pattern	CSNL	B-cell lymphoblastic leukemia involving CNS
13	25	M	ALL (T cell)	Headache, Seizures, Limb Weakness, Decreased vision in both eyes	There is a mass lesion in the right hemisphere of the brain, with mild enhancement around the lesion and ventricular dilation	CSNL	T-cell lymphoblastic leukemia involving CNS
14	60	M	CMML	Nausea, Vomiting, Right-sided limb weakness, Altered consciousness	A 2.5 × 3 cm mass lesion is visible in the left basal ganglia and thalamus, protruding into the left ventricle and invading the corpus callosum. The left ventricle is deformed due to compression, with significant enhancement of the lesion	CSNL	Chronic myeloid leukemia involving CNS
15	26	M	ALL(B cell)	Headache	Multiple lesions are observed in the left temporal lobe, accompanied by surrounding brain tissue edema and meningeal enhancement	CSNL	B-cell lymphoblastic leukemia involving CNS
16	29	F	AML(M5)	/	Abnormal mass-like signals are present in both cerebral hemispheres, with abnormal enhancing signals observed in both cerebellar hemispheres and the left occipital lobe	CSNL	Myeloid leukemia involving CNS
17	42	M	ALL(B cell)	Dizziness, Vomiting, Unsteady gait	Nodular enhancing lesions are visible in the vermis of the cerebellum, the roof of the fourth ventricle, and the left cerebellar hemisphere	CSNL	B-cell lymphoblastic leukemia involving CNS
18	39	M	ALL(B cell)	Headache	There is a slightly long T1 and short T2 lesion in the right cerebellar hemisphere, which enhances after contrast administration. The enhancement is uneven, and the border is unclear. The size of the mass is approximately 6 cm	CSNL	B-cell lymphoblastic leukemia involving CNS
19	26	M	ALL(B cell)	Headache	Multiple mass-like lesions with slightly long T1 and slightly short T2 signals are visible in the left temporal lobe, with small punctate short T1 signals within. There is significant surrounding edema extending to the thalamus and basal ganglia. The lesions exhibit marked enhancement, which is uneven, with slightly higher enhancement along the edges	CSNL	B-cell lymphoblastic leukemia involving CNS
20	29	M	ALL(T cell)	Headache	There are long T1 and long T2 signals in the left temporal lobe and the lateral wall of the left orbital cavity, with uneven enhancement. The enhanced portion of the left temporal lobe exhibits peripheral low signal. The left optic nerve is compressed	CSNL	T-cell lymphoblastic leukemia involving CNS
21	7	M	ALL(B cell)	/	There are multiple thickened lesions in the midbrain, pons, and medulla oblongata. Nodular enhancement is evident in the central part of the pontine lesion, with mild linear enhancement observed around it	CSNL	B-cell lymphoblastic leukemia involving CNS
22	32	M	AML(M5)	Dizziness, Headache	There are abnormal nodular or patchy masses in the right temporal lobe and the left cerebellar hemisphere. These masses exhibit short T1 and long T2 signals and show uniform enhancement. There is significant surrounding brain tissue edema	CSNL	Myeloid leukemia involving CNS

*CSNL* leukemia involving the central nervous system, *ALL* acute lymphoblastic leukemia, *AML* acute myeloid leukemia, *CLL* chronic lymphoblastic leukemia, *CMML* chronic myelomonocytic leukemia, *CNS* central nervous system

### Pathological diagnosis

**Fig. 1:**
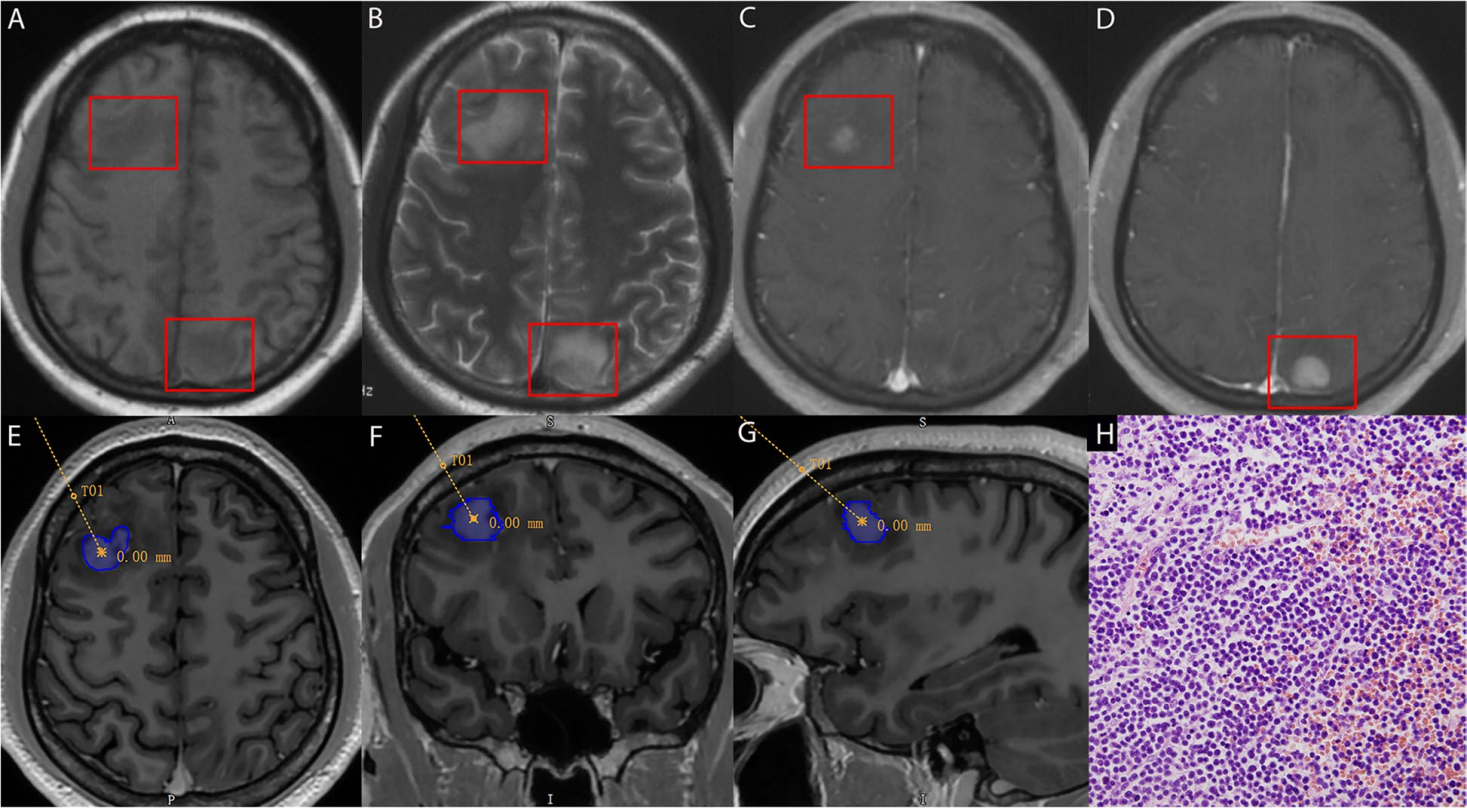
MRI (**A**,** B**,** C**,** D**), preoperative biopsy plan (**E**,** F**,** G**), and pathological results (**H**) of a 22-year-old male patient with acute lymphoblastic leukemia (ALL). MRI (**A**,** B**,** C**,** D**, red rectangular boxes) indicates multiple patchy and nodular abnormal signals (**A,** T1) in the right frontal–parietal lobe (biopsy site), left parieto–occipital cortex, and subcortex, with significant enhancement (**C **and **D**, enhanced) and marked perilesional edema (**B**, T2). Pathological results show diffusely dense tumor cell growth. The cells are small, with scant cytoplasm, coarse nuclear chromatin, and frequent mitotic figures. Immunohistochemistry results: CD20 ( −), CD79a ( +), CD3 (T lymphocytes +), CD5 (T lymphocytes +), CD30 ( −), BCL-6 (slightly +), CD10 ( +), MUM-1 ( −), PAX-5 ( +), Cyclin D1 ( −), BcL-2 ( +), P53 (slightly +), c-myc (partially +), EBV (in situ hybridization) ( −), CD34 (vascular +), CD99 ( −), GFAP (brain tissue +), TdT ( +), Syn ( −), IgG4 ( −), and Ki-67 (95% +). MRI, Magnetic Resonance Imaging; BCL-6, B-cell Lymphoma Gene; MUM-1, Multiple Myeloma Oncogene 1; PAX-5, Paired Box 5; BCL-2, B-cell Lymphoma 2; P53, Tumor Protein 53; c-myc, Cellular Myelocytomatosis; EBV, Epstein-Barr Virus; GFAP, Glial Fibrillary Acidic Protein; TdT, Terminal Deoxynucleotidyl Transferase; Syn, Synaptophysin; IgG4, Immunoglobulin G4

**Fig. 2:**
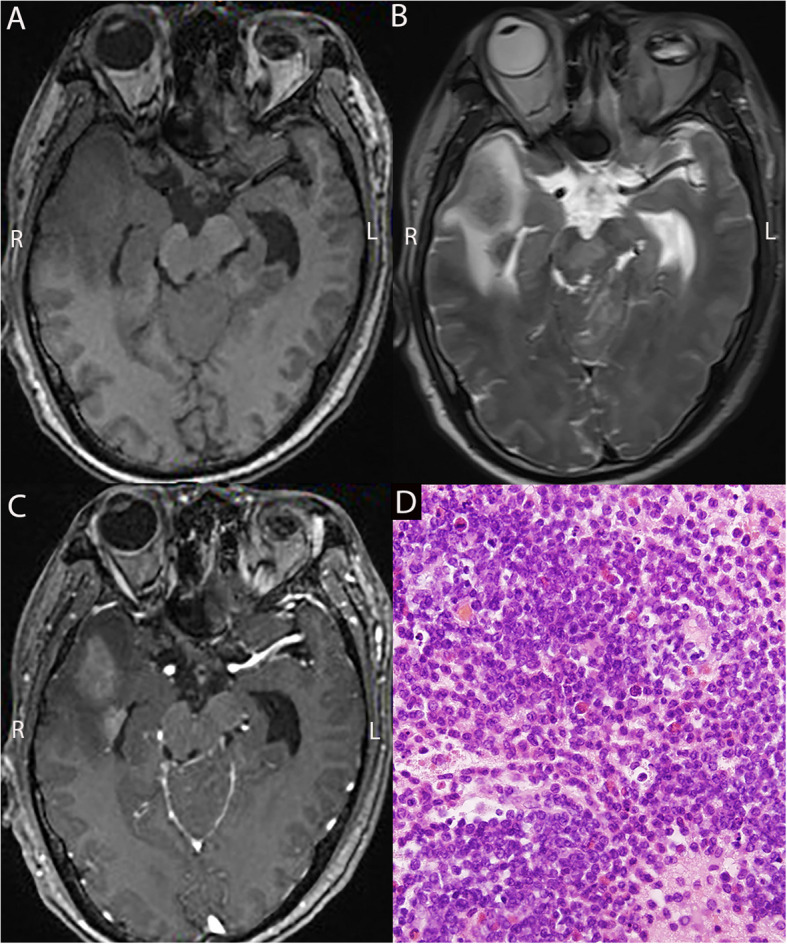
Preoperative MRI (**A**,** B**,** C**) and pathological results (**D**) of a 22-year-old male patient with acute myeloid leukemia (AML). MRI indicates an abnormal nodular mass in the right temporal lobe (biopsy site) and left cerebellar hemisphere, with short T1 (**A**), long T2 (**B**), and homogeneous enhancement (**C**). Significant edema is observed in the surrounding brain tissue. Pathological results show proliferative lymphocytes in the brain tissue; some are arranged in perivascular lymphocytic cuffing structures. The cells exhibit atypia with dense growth of small tumor cells and frequent mitotic figures. Immunohistochemistry results: CD20 ( −), CD3 ( −), CD5 (a few +), MPO ( +), CD34 ( +), CD117 ( +), CD38 ( +), TdT ( −), and Ki-67 (70% +). MRI, Magnetic Resonance Imaging; MPO, Myeloperoxidase; TdT, Terminal Deoxynucleotidyl Transferase

Pathological diagnoses of the CNS lesions were obtained using stereotactic intracranial biopsy (Table 1). Among the 22 patients, 13 had leukemia involving the CNS (CNSL) (nine cases of ALL, three of AML, and one of CMML; Fig. 1H and Fig. 2D). Of the CNSL cases, one occurred during chemotherapy, seven occurred during the complete remission period after chemotherapy, and five occurred after allogeneic hematopoietic stem cell transplantation (allo-HSCT).

**Fig. 3:**
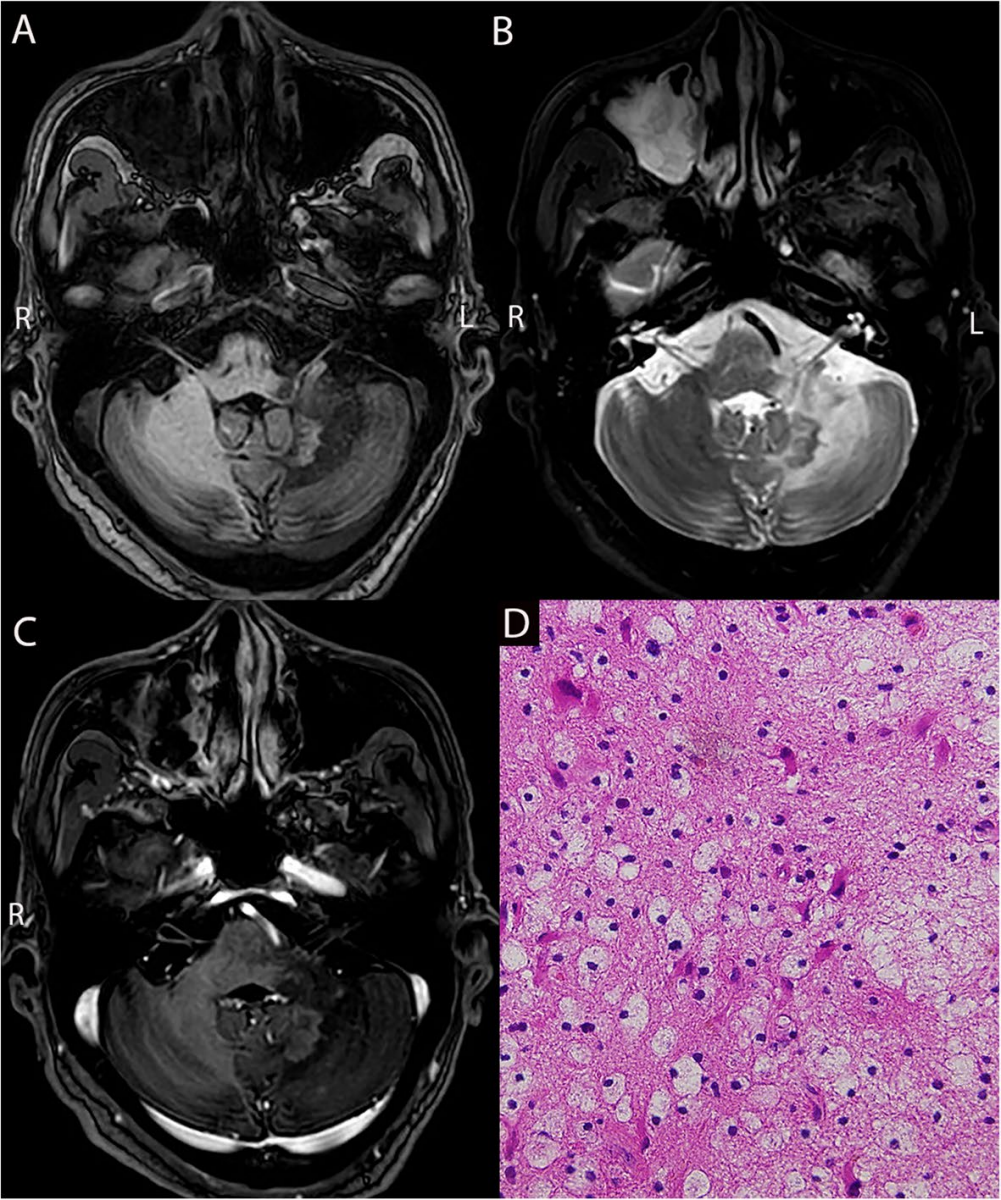
MRI (**A**,** B**,** C**) and pathological results (**D**,** E**) of a 39-year-old male patient with acute lymphoblastic leukemia (ALL). MRI showed abnormal mixed patchy signals adjacent to the right temporal lobe ventricle (puncture biopsy site), with mixed T1 (**A**) and long T2 signals (**B**), surrounding brain tissue edema, and partial enhancement (**C**). Multiple abnormal signals are observed adjacent to the left lateral ventricular posterior horn, with small circular volumes, short T1 (**A**) and long T2 signals (**B**), and surrounding brain tissue edema with ring enhancement (**C**). Pathologically (**D**), abundant fungal hyphae are observed between cells, appearing relatively uniform and possibly sharply branched. Silver staining (**E**) confirms the presence of fungi; Giemsa staining ( −) and acid-fast staining ( −) yielded negative results. Immunohistochemistry results: GFAP (+ + +), Olig2 ( +), P53 (negative), Ki67 (approximately 2% positive), LCA ( +), CD3 (+ + +), CD20 (negative), and CD68 (+ + +). MRI, Magnetic Resonance Imaging; Olig2, Oligodendrocyte transcription factor 2; P53, Tumor Protein 53; GFAP, Glial Fibrillary Acidic Protein; LCA, Leukocyte Adhesion Deficiency

The nine patients with CNS secondary lesions related to leukemia treatment included five cases of CNS infection (three of fungal brain abscesses, one of brain abscess of unknown etiology, and one of tuberculoma; Fig. 3D) and four of neurodegenerative lesions (two of demyelinating lesions and two of gliosis). Among the five cases with CNS infections, three occurred during immunosuppressive therapy (cyclosporine and/or anti-thymocyte globulin) following allo-HSCT, and two occurred during the second and fourth chemotherapy courses. Of the two patients with demyelinating lesions, one developed demyelination after three courses of systemic chemotherapy and five courses of intrathecal chemotherapy (Fig. 3D and E); the other developed demyelination during the complete remission period after chemotherapy. Of the two patients with gliosis, one developed gliosis approximately 1 month after high-dose chemotherapy and allo-HSCT; the other developed gliosis during the complete remission period after chemotherapy.

In eight patients, the clinical diagnosis of CNS lesions did not match the pathological diagnosis (Cases 2, 3, 4, 5, 6, 7, 10, and 11). The clinical diagnosis for Cases 2, 4, 5, 6, and 7 was CNSL; however, the pathological diagnoses were fungal brain abscess, brain abscess of unknown etiology, tuberculoma, and gliosis (two cases). The clinical diagnosis in Case 3 was neurodegenerative disease, but the pathological diagnosis was a fungal brain abscess. The clinical diagnoses for Cases 10 and 11 were neurodegenerative disease and brain abscess, respectively, whereas the pathological diagnosis was CNSL.

### Imaging features

Cranial CT scans of 13 patients with CNSL showed patchy low-density shadows in the brain parenchyma. Cranial MRI scans of these patients typically revealed slightly prolonged or isointense T1 and prolonged T2 signals in the lesion areas. All lesions exhibited significant enhancement. Among the cases, 12 patients presented with nodular enhancement (eight with multiple lesions and four with a single lesion), and one patient showed diffuse patchy enhancement (all multiple lesions). In the three cases of fungal brain abscesses, MRI showed scattered, multiple small cystic lesions with short/prolonged T1 signals, prolonged T2 signals, unclear boundaries, and significant perilesional edema (Figs. 3A–C). One case of brain abscess with an unknown pathogen and one case of tuberculoma presented as single lesions with mixed signals, significant enhancement, and obvious perilesional brain tissue edema.

**Fig. 4:**
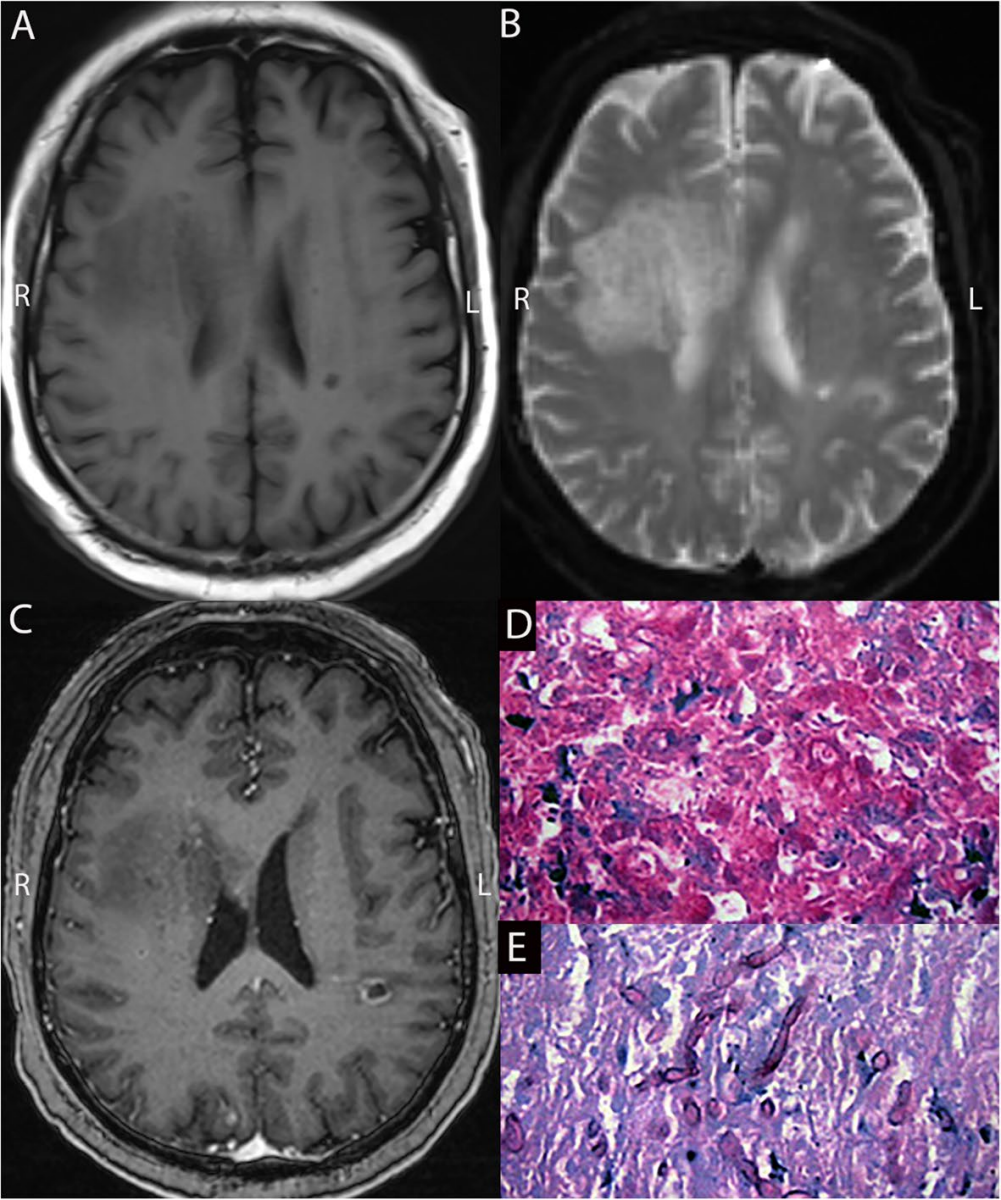
Preoperative MRI (**A**,** B**,** C**) and pathological results (**D**) of a 60-year-old male patient with chronic lymphoblastic leukemia (CLL). The MRI indicates patchy abnormal signals in the left pontine and cerebellar hemispheres and around the bilateral lateral ventricles, with short T1 (**A**) and long T2 (**B**) signals and no mass effect. Only partial enhancement is observed in the lesions in the left pontine and cerebellar hemispheres (**C**). Pathology (**D**) showed a small amount of brain tissue with numerous lymphocytic infiltrates, focal lymphocytic infiltration around the blood vessels, and fresh focal bleeding. Immunohistochemical staining reveals demyelination with relatively preserved axons and some cells with large, deeply stained nuclei; close follow-up is recommended. Immunohistochemistry results: GFAP ( +), MBP (partially +), CD3 (few T lymphocytes +), CD20 (few B lymphocytes +), CD68 (macrophages +), Ki-67 (individual +), Olig2 (few astrocytes +), and LFB + HE (demyelination). Demyelinating lesions are considered in this case. MRI, Magnetic Resonance Imaging; Olig2, Oligodendrocyte transcription factor 2; GFAP, Glial Fibrillary Acidic Protein; MBP, Myelin Basic Protein; LFB, Luxol Fast Blue; HE, Hematoxylin & Eosin

Among the four patients with neurodegenerative lesions, the MRI scans of those with demyelinating lesions showed diffuse patchy lesions without significant mass effects. One patient showed partial enhancement (Figs. 4C); the other showed no enhancement. Patients with gliosis exhibited significant mass effects and marked perilesional edema. One patient had multiple significantly enhanced lesions on MRI; the other had a single ring-enhanced lesion with no internal enhancement.

## Discussion

LCL primary involves leukemia cells infiltrating the CNS, as well as CNS secondary lesions related to leukemia treatment, including demyelinating lesions, infections, secondary tumors, and cerebrovascular-related lesions [4, 5]. CNSL refers to extramedullary leukemia resulting from leukemic cells infiltrating the brain parenchyma, cranial nerves, meninges, and spinal cord. In clinical practice, CNSL often occurs during remission after chemotherapy. Despite significant progress in understanding the pathogenesis, diagnosis, and treatment of leukemia over the past few decades, CNSL remains one of the most serious complications and a major cause of mortality. CNS involvement occurs in approximately 3–5% of newly diagnosed patients and in approximately 30–40% of relapsed cases [10, 11]. Early autopsies show that most patients with leukemia develop CNSL during treatment [10–12, 21]. All leukemia types can cause CNSL, with the highest incidence observed in ALL, which is more common in children than in adults [10–12, 22, 23]. The present study validated this finding; among the 13 CNSL cases, nine were ALL (69.23%), three were AML (23.08%), and one was CMML (7.69%).

The invasion of leukemia cells into the CNS is related to several factors. A subset of leukemia cells, known as adhesive cells, express various adhesion molecules (including very late antigen-4 [VLA-4], intercellular adhesion molecule-1 [ICAM-1], cluster of differentiation 18 [CD18], lymphocyte function-associated antigen-1 [LFA-1], cluster of differentiation 58 [CD58], cluster of differentiation 44 [CD44], and vascular cell adhesion molecule [VCAM]), facilitating adhesion to vascular endothelial cells, leukocyte stasis in the vasculature, and promoting tumor growth and angiogenesis [11, 24].

Leukemia stem cells (LSCs) are an important leukemia cell subset that exhibits strong adaptability to new microenvironments, enabling them to resist adverse environmental factors. In addition, LSCs often overexpress cluster of differentiation 56 (CD56) on their surface, enhancing their ability to enter the CNS. Therefore, leukemia cells, especially LSCs, can migrate along vascular channels connecting the cranial and spinal bone marrow to the dura mater and penetrate through the vascular endothelium via mechanisms including transendothelial migration, diffuse infiltration of the meninges, migration in the cerebrospinal fluid, and rapid proliferation, further infiltrating the perivascular spaces and brain parenchyma. Moreover, a hypoxic microenvironment coupled with the secretion of vascular endothelial growth factor-A (VEGF-A) by leukemia cells increases vascular permeability and BBB disruption, making the CNS more susceptible to invasion. The immunologically privileged nature of the CNS protects leukemia cells from the cytotoxic effects of chemotherapy [11, 24].

The imaging characteristics of CNSL lack specificity; CT often showed patchy, low-density shadows in the brain parenchyma. On MRI, most lesions appear as multiple scattered lesions within the brain parenchyma. However, single lesions can also be observed with clear demarcation from the normal brain tissue, severe local edema, and generally equal or slightly longer T1 and T2 signals with significant enhancement [4, 11, 25]. In this study, the pathological manifestations of the 13 cases of CNSL were diverse, leading to complex and variable imaging findings. Among them, 12 patients showed nodular infiltration of tumor cells in the brain parenchyma as the main pathological feature, presenting as multiple or solitary, scattered, round, solid nodules in the brain parenchyma, with lesions on MRI appearing as slightly long or equal T1 and long T2 signals with significant surrounding edema and marked enhancement. One patient showed tumor cell infiltration predominantly around brain meningeal vessels; MRI showed rim-like enhancement of the meninges and local meningeal thickening. Another patient showed tumor cell infiltration that blocked the arterial and venous blood supply and brain parenchyma drainage; MRI showed small local infarctions, ischemic or hemorrhagic foci, venous stasis-related brain edema, or hemorrhage.

Differentiating CNSL from CNS infections and neurodegenerative diseases based on clinical symptoms and imaging examinations can be challenging [5, 6]. In this study, nine patients were diagnosed with CNS infections or neurodegenerative diseases, including fungal brain abscesses (*n* = 3), brain abscesses (one patient with unidentified pathogens), tuberculomas (*n* = 1), demyelinating lesions (*n* = 2), and gliosis (*n* = 2), all of which were secondary CNS lesions related to leukemia treatment. These conditions may occur after receiving high-dose chemotherapy, steroids, immunosuppressants, or radiotherapy to the CNS. Four patients developed CNS infections (brain abscesses and tuberculomas) due to immunodeficiency following high-dose chemotherapy during bone marrow transplantation or after receiving immunosuppressive therapy for allo-HSCT.

Currently, the gold standard for diagnosing CNSL includes a morphological examination of the cerebrospinal fluid (CSF), which has high specificity but relatively low sensitivity, often leading to false-negative results and necessitating a more accurate diagnostic method [12, 26]. Flow cytometry analysis of the CSF aids in diagnosing CNSL, with greater sensitivity than that of a morphological examination [9]. However, flow cytometry has not been widely adopted because of various constraints; for example, the number of antibodies in the CSF limits the technique.

The imaging features of CNSL lack specificity, and the difficulties and uncertainties associated with CSF analysis can delay diagnosis and treatment of CNSL. Misdiagnosis rates of CNSL can be as high as 75%; CNSL is often misdiagnosed as intracranial hemorrhage, cerebral infarction, meningitis, infection, demyelination, multiple sclerosis, spinal cord compression, or Bálint syndrome [7, 8]. Clinical misdiagnosis and empirical treatment can delay the optimal timing of treatment initiation, significantly contributing to disease progression, greatly reducing the quality of life, and shortening the survival of patients with CNSL [6, 7, 9, 11, 22].

Developments in neuroimaging technology, neurosurgical navigation techniques, and surgical robots in clinical practice have improved visualization, automation, and precision in stereotactic intracranial lesion biopsies. Intraoperative histopathological examination is the gold standard for diagnosing intracranial lesions. Although it poses a greater risk for CSF sampling than does than lumbar puncture, its accuracy is far superior to that of morphological examination and analysis of the CSF [6, 11]. Stereotactic intracranial lesion biopsy is minimally invasive, safe, and easy to perform [14, 19]. Therefore, the diagnostic rate can be improved by combining routine examinations, including medical history assessment, hematological tests, bone marrow puncture, routine and biochemical CSF analyses, and stereotactic intracranial lesion biopsy imaging studies [27]. Early diagnosis facilitates the correct formulation of timely treatment plans and helps avoid unnecessary treatments and related mortality. Therefore, applying stereotactic intracranial lesion biopsy can expedite the diagnosis of CNSL, increase its accuracy [6, 11], achieve standardized and precise treatment, improve patients’ quality of life, and prolong survival. Pathological diagnoses in this study were made through stereotactic intracranial lesion biopsy in all 22 patients with LCL; no surgical complications occurred postoperatively.

Stereotactic biopsy, as a minimally invasive surgical technique, has demonstrated high accuracy and safety in the diagnosis of central nervous system (CNS) lesions. Studies have shown that this procedure requires only a small burr hole in the skull, thereby avoiding the trauma and prolonged recovery time associated with craniotomy [28, 29]. In terms of safety, the overall complication rate reported in the literature is low. One study documented a postoperative complication rate of only 4.47%, primarily involving minor postoperative bleeding, with no severe consequences [30, 31]. Intracranial hemorrhage is the most common serious complication of stereotactic brain biopsy, with a reported incidence of approximately 0.5%–3% in previous studies [16, 17]. Stereotactic biopsy, owing to its minimally invasive nature, low surgical risk, and high procedural efficiency, has become a crucial technique for the histological diagnosis of CNS lesions, facilitating improved perioperative management for patients. However, its potential complications must be carefully considered to achieve a balance between diagnostic accuracy and procedural safety.

After being diagnosed with LCL, patients undergo appropriate treatment while being closely monitored throughout the process. Monitoring includes two key aspects: (1) Imaging evaluations, with a cranial CT scan performed within 24 h post-biopsy to assess for acute complications such as hemorrhage, followed by MRI scans at three months, six months, one year, and two years to detect lesion progression or recurrence; (2) CSF-based assessments, where flow cytometry or circulating tumor DNA analysis is conducted alongside imaging follow-ups to monitor for minimal residual disease, early recurrence, or new CNS involvement.

In summary, with continuous improvements in anti-leukemia treatment efficacy and patient survival, the incidence of LCL is increasing. The typical imaging features of CNSL include multiple scattered round solid nodules in the brain parenchyma with slightly elongated or equal T1 and long T2 signals. However, distinguishing CNSL from CNS secondary lesions is crucial because of the lack of specificity in imaging findings and the challenges and uncertainties associated with CSF analysis. This study is limited by its retrospective single-center design, relatively small sample size and lack of direct comparison with alternative diagnostic methods, highlighting the need for future multicenter, prospective studies to further validate and expand upon our findings.

## Conclusion

In this study, the clinical diagnoses were inconsistent with the pathological diagnoses in eight patients. Appropriate treatment strategies can achieve good therapeutic effects in most cases of LCL. Stereotactic intracranial lesion biopsy is minimally invasive and has high safety and surgical convenience. It plays an extremely important role in the early and differential diagnosis of LCL. Early determination of lesion nature and timely adoption of correct and precise treatment strategies can improve patient prognosis.

## Data Availability

All the data in this study were obtained from the corresponding author for reasonable reasons.

## Abbreviations

ALL: Acute lymphoblastic leukemia
allo-HSCT: Allogeneic hematopoietic stem cell transplantation
AML: Acute myeloid leukemia
BBB: Blood-brain barrier
BCL-2: B-cell Lymphoma 2
BCL-6: B-cell Lymphoma Gene
CD18: Cluster of Differentiation 18
CD44: Cluster of Differentiation 44
CD56: Cluster of Differentiation 56
CD58: Cluster of Differentiation 58
CLL: Chronic lymphoblastic leukemia
CMML: Chronic myelomonocytic leukemia
c-myc: Cellular Myelocytomatosis
CNS: Central nervous system
CSF: Cerebrospinal fluid
CT: Computed Tomography
EBV: Epstein-Barr Virus
GFAP: Glial Fibrillary Acidic Protein
HE: Hematoxylin & Eosin
ICAM-1: Intercellular Adhesion Molecule-1
IgG4: Immunoglobulin G4
LCA: Leukocyte Adhesion Deficiency
LFA-1: Lymphocyte Function-Associated Antigen-1
LFB: Luxol Fast Blue
LSCs: Leukemia stem cells
MBP: Myelin Basic Protein
MPO: Myeloperoxidase
MRI: Magnetic Resonance Imaging
MUM-1: Multiple Myeloma Oncogene 1
Olig2: Oligodendrocyte transcription factor 2
P53: Tumor Protein 53
PAX-5: Paired Box 5
PET: Positron Emission Tomography
Syn: Synaptophysin
TdT: Terminal Deoxynucleotidyl Transferase
VCAM: Vascular Cell Adhesion Molecule
VEGF-A: Vascular Endothelial Growth Factor-A
VLA-4: Very Late Antigen-4
